# A Personal Tribute to a Highly Inspiring Mentor, Professor Sir Peter J. Lachmann, 1931–2020

**DOI:** 10.3390/v13020206

**Published:** 2021-01-29

**Authors:** Reinhard Würzner

**Affiliations:** Institute of Hygiene and Medical Microbiology, Medical University of Innsbruck, Schöpfstraβe 41, A-6020 Innsbruck, Austria; reinhard.wuerzner@i-med.ac.at

With great sadness and sympathy for his family, especially for his wife and companion for many decades, Sylvia, I convey the news of the peaceful passing of Professor Sir Peter Lachmann on 26 December 2020, three days after his 89th birthday and after a Christmas celebration—as far as this was possible these days—in the heart of his family.

I am conveying my deep feelings on behalf of the European Complement Network, whose Gold Medal for life time achievements in the field of complement he earned as one of the first in 1997, as its president, and refer to a short obituary together with the International Complement Society, ICS on the ECN homepage [1]. For Peter’s appointments and honors I refer to obituaries by his college [2] and by Complement UK [3]. For another very personal tribute on his influence on the complement field, and speaking for the International Complement Society, I refer to an obituary by Richard Harrison and Paul Morgan, to be published in Focus, the international newsletter for complementologists [4].

Here I will present a very personal tribute, not focusing on Peter as the titan of complement, but on his role as my mentor, elucidating some personal views, from the way I became Peter’s proud PhD student to (only one of) his last scientific role(s) as a Guest Editor of the Special Issue on “Viruses and Complement” for MDPI–Viruses and how this was inaugurated.

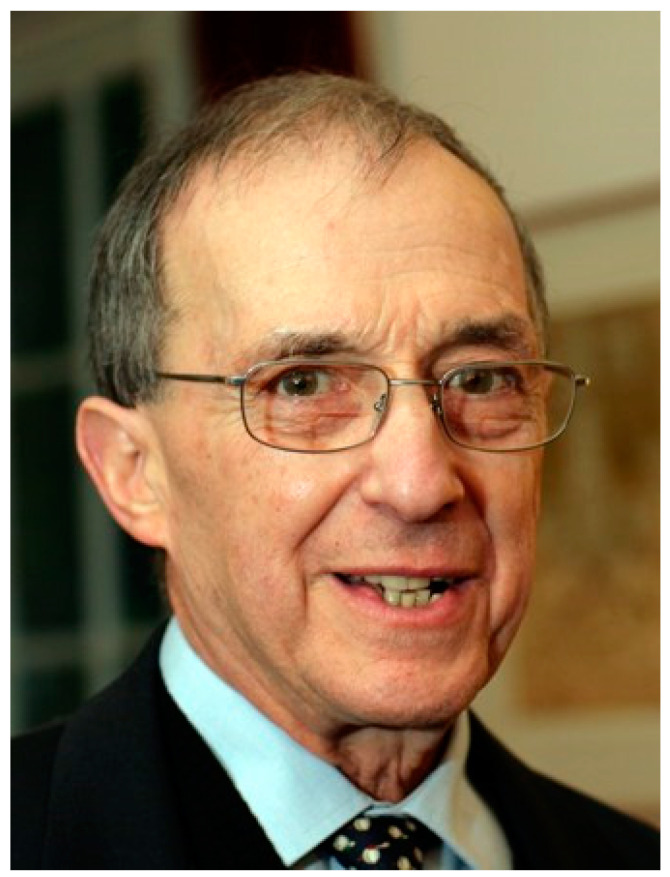



Professor Sir Peter J. Lachmann, 1931–2020 (Courtesy of Christ’s College, Cambridge).

When I entered the field, face-to-face at the XII International Complement Workshop in Chamonix in 1987, having read already a lot from the several distinguished professors present then, I became immediately aware of an extremely bright scientist who was clearly different: always sitting in the first row, never missing a session (I skipped two to see the beautiful Mont Blanc glacier), always able to comment on the different facets of complement and something that helped us young scientists a lot: whereas other scientists simply nodded or remained silent, he always spoke up when something had to be looked at from a different angle and why, and noting that this finding had already been discovered in the past. This was not always pleasant for the presenter, but it helped us youngsters a lot to learn what is right or wrong, to be critical and to get a structure into this not too easy to understand cascade system.

From that meeting, it was clear to me that I had to ask him (done at the next complement conference in Bari 1988)—and nobody else—to become my future PhD supervisor. At that time I was working as an MD in complement research in Göttingen with Otto Götze. As a side effect, I also considered it helpful not to have Peter against me in future then, :-).

From 1990 till 1993 I had a wonderfully productive and stimulating time in Cambridge as Peter’s PhD student—I always refer to that time as the best in my life. This was of course also due to the exceptional team he had at that time, Mike Hobart, Barbara Fernie and Ann Orren with whom I had the privilege to learn and study complement, especially C6 and C7. 

One of my topics was to study the C7 complement polymorphism based on a monoclonal antibody and when he called me into his office early one day, he suggested looking at liver transplanted subjects before and after the transplantation to see whether the allotype of C7 would change (I had many other discussions with Peter where it was more difficult to follow his bright ideas and trains of thought). From the finding well-known to him, but not to me, that C6 was an acute phase protein, but not C7, he hypothesized that C7 is probably not exclusively produced in the liver, as the majority of the other complement proteins. Peter was of course right ([5], one out of 20 papers with Peter) and the reactive lysis mechanism, he discovered with Ron Thompson earlier [6] now became even more important: C7 became a local modulator of complement attack and—irrespective of the presence or absence of other inhibitors, complement membrane attack would proceed, simply if C7 is contributed locally, and impaired (or inhibited if you like), if no C7 is locally provided [7]. 

Two seminal things happened in the world in mid-September, 1991; the iceman was found in the (South!) Tyrolean alps and Peter hosted the XIVth International Complement Workshop in Cambridge. He was actually very proud to do that and we were proud to be part of his team. Apart from the scientists mentioned above, I would like to mention his administrator, secretary and right hand, Sarah Coppendale, the heart of the organization committee (and who also helped with personal things, such as accommodation)—she was praised by one of the delegates as Peter’s “most prominent secretary”—and, of course, Dick Harrison, who later become a much closer friend. This workshop was different as we (!) did not hold it in a big conference hall, but in the many colleges, providing a very personal charm. Peter’s belief in a young active scientist was also strong when it came to delegating important issues. I was appointed responsible for the poster sessions, and actually from then onwards, I often got this task at conferences—his guidance and influence was not often obviously seen. 

An obituary for Peter would not be complete without reference to him as family man. Dick Harrison and Paul Morgan stated in their obituary that “not only did he have an encyclopaedic memory of science, he sometimes seemed to remember more about your own family than you did yourself”—this is an excellent description and the only thing one has to add is that this also involved Sylvia, his companion for many decades. She was always a stronghold for Peter—sometimes even physically, as in a late night encounter in Bari when she chased away some muggers—and I would say that she was sort of his “alter ego”, fully independent with her interesting archeology studies, but still likely his most important critic—who else could criticize this giant? I very much enjoyed the stays at their house, the books I would read before going to sleep and the breakfast with Peter, still in his morning gown, when the morning sun was shining through the beautiful Victorian leaded glass windows and the delicious English breakfast with the clearly too dark toast.

Many friends of Peter (and Sylvia) could keep in contact with them due to their lively annual newsletters they used to send by mail and later by email shortly before Christmas. I always wanted to write to both in time for Christmas as well (with Peter’s birthday on the 23rd as another reason), but I never managed in time. It was easy to keep in touch with Peter (and Sylvia), also because they continued to attend complement meetings and the last meeting with Peter was probably the Complement UK meeting in March 2019.

About a year ago Peter approached me and asked me whether I would be his Co-Guest Editor for a Special Issue of “Viruses and Complement” for MDPI–Viruses (this issue). I felt very honored and privileged that he thought of me and I enjoyed working closely with him as well. “Mens sana in corpore sano” unfortunately subsided in the last years, but clearly on the second part of the expression. So it was inspiring as ever before to discuss science with him. During the work as Guest editor I am now confronted with the sad fact that I cannot talk to him anymore, on an almost daily basis. But I had never stopped thinking of him anyway. 

Complement has lost a giant—I have lost my mentor and fatherly friend and my thoughts are with his family and especially with Sylvia.

Reinhard Würzner, 

Inst. Hyg. Med. Microbiology, Med. Univ. Innsbruck, Austria

PhD Student in Cambridge with Sir Peter Lachmann, 1990–1993

President of the European Complement Network

Guest Editor with Peter Lachmann for “Viruses and Complement” in MDPI-Viruses.
